# ecc_finder: A Robust and Accurate Tool for Detecting Extrachromosomal Circular DNA From Sequencing Data

**DOI:** 10.3389/fpls.2021.743742

**Published:** 2021-12-01

**Authors:** Panpan Zhang, Haoran Peng, Christel Llauro, Etienne Bucher, Marie Mirouze

**Affiliations:** ^1^Institut de Recherche pour le Développement (IRD), Montpellier, France; ^2^Laboratory of Plant Genome and Development, University of Perpignan, Perpignan, France; ^3^Crop Genome Dynamics Group, Agroscope Changins, Nyon, Switzerland; ^4^Department of Botany and Plant Biology, Section of Biology, Faculty of Science, University of Geneva, Geneva, Switzerland; ^5^Laboratory of Plant Genome and Development, Centre National de la Recherche Scientifique (CNRS), Perpignan, France

**Keywords:** eccDNA, nanopore amplicon sequencing, mobilome, *Arabidopsis*, wheat

## Abstract

Extrachromosomal circular DNA (eccDNA) has been observed in different species for decades, and more and more evidence shows that this specific type of DNA molecules may play an important role in rapid adaptation. Therefore, characterizing the full landscape of eccDNA has become critical, and there are several protocols for enriching eccDNAs and performing short-read or long-read sequencing. However, there is currently no available bioinformatic tool to identify eccDNAs from Nanopore reads. More importantly, the current tools based on Illumina short reads lack an efficient standardized pipeline notably to identify eccDNA originating from repeated loci and cannot be applied to very large genomes. Here, we introduce a comprehensive tool to solve both of these two issues.^1^ Applying ecc_finder to eccDNA-seq data (either mobilome-seq, Circle-Seq and CIDER-seq) from *Arabidopsis*, human, and wheat (with genome sizes ranging from 120Mb to 17 Gb), we document the improvement of computational time, sensitivity, and accuracy and demonstrate ecc_finder wide applicability and functionality.

## Introduction

**Figure 1:**
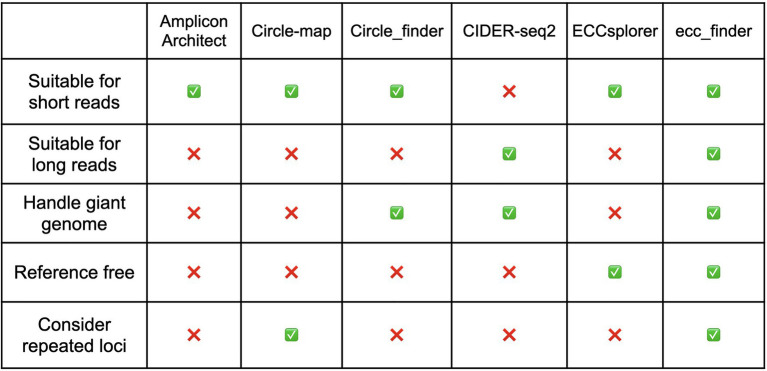
Summary of the characteristics of up-to-date eccDNA detection tools.

Circular DNA is a ubiquitous form of biological DNA molecules. Indeed, it can be found as bacterial, viral, mitochondrial, and chloroplastic genomes and plasmids, but also as extrachromosomal circular DNA (eccDNA) in eukaryotes (Hotta and Bassel, 1965). eccDNA has been described for decades in yeasts (Sinclair and Guarente, 1997), *Drosophila* (Cohen et al., 2003), mammals (Cohen et al., 2006; Kumar et al., 2017), and plants (Hirochika and Otsuki, 1995). Recently, the role of eccDNA as an important genomic feature of cancer cells has been revealed. Indeed, in cancer cells, eccDNA molecules arise from chromosomal oncogenes inducing their overexpression (Kumar et al., 2017) and are associated with poor prognosis (Verhaak et al., 2019; Kim et al., 2020; Wang et al., 2021) and drug resistance (Yan et al., 2020). In plants, genes located in eccDNA molecules can be overexpressed leading to herbicide resistance (Koo et al., 2018). Besides genes, eccDNA can arise from repetitive genomic sequences, such as telomeric DNA (Cohen and Méchali, 2002; Zellinger et al., 2007; Mazzucco et al., 2020), satellites (Navrátilová et al., 2008), or ribosomal RNA genes (rDNA; Sinclair and Guarente, 1997) through homologous recombination. Moreover, eccDNA is part of the life cycle of certain types of active transposable elements (TEs; Hirochika and Otsuki, 1995; Lanciano et al., 2017). The presence of eccDNA thus generally reflects genome plasticity. We previously developed Mobilome-seq (Lanciano et al., 2017, 2021) as a method to selectively sequence eccDNA purified from plants or animal tissue. The method is based on two main steps: (1) linear DNA digestion using an ATP-dependent DNase followed by (2) eccDNA enrichment by random rolling circle amplification. Several similar methods have been established to enrich and detect eccDNA molecules, such as Circle-Seq (Møller et al., 2015) and CIDER-seq (Mehta et al., 2020). With the arrival of single-molecule real-time sequencing by Pacific Biosciences and nanopore sequencing by Oxford Nanopore Technologies (ONT), eccDNA sequencing with long reads allows capturing comprehensive eccDNA content by spanning the full length of eccDNA in one read (Koche et al., 2020). However, following short- or long-read sequencing, only a handful of bioinformatic tools has been developed for the downstream analysis of eccDNA data. CIDER-Seq2 is the only tool based on PacBio long reads alone. AmpliconArchitect (Deshpande et al., 2019), Circle-Map (Prada-Luengo et al., 2019), Circle_finder (Kumar et al., 2017), and ECCsplorer^2^ are tools based on Illumina short reads. Moreover, except for ECCsplorer, all software packages require a reference genome, thus limiting the analyses to model species (Figure 1).

Here, we developed a new tool called ecc_finder dedicated to the detection of eccDNA from both Illumina and Nanopore eccDNA sequencing data. We demonstrate its suitability and sensitivity when applied on eccDNA data sets originating from small (*Arabidopsis thaliana,* 120Mb) and very large genomes (wheat *Triticum aestivum,* 17 Gb) for detecting eccDNAs.

## Materials and Methods

### Description of the ecc_finder Algorithm and Validation Metrics

The complete ecc_finder source code and documentation are available on GitHub at https://github.com/njaupan/ecc_finder. ecc_finder is written in Python3 and relies on two mapping tools: minimap2 (Li, 2018) for ONT long reads and BWA (Li and Durbin, 2009) for Illumina short reads. It also bundles TideHunter (Gao et al., 2019) for discovering tandem repeat patterns and generating high-quality consensus sequences and Genrich^3^ for peak calling. ecc_finder mainly uses the PAF format generated by paftools.js to realize format conversion and alignment filtering.

### Long-Read Pipeline Algorithm Overview

Thanks to the Phi29 rolling circle amplification of eccDNAs, the matrix for long-read sequencing comprises tandem repeats of the original eccDNA sequence. Therefore, reads originating from circular DNA will display two or more sub-read alignments to the reference in the same direction. ecc_finder thus uses a tandem repeat pattern detection from read alignments to identify candidate loci. First, to exclude long reads originating from linear genomic repeats (such as satellites), ecc_finder performs standard alignment to extract alignment block length using minimap2. By default, ecc_finder will remove any alignment shorter than 200bp (Supplementary Figure 4). ecc_finder then uses TideHunter to identify candidate reads with a tandem repeat pattern and divide each read into repeat units (Supplementary Figure 4). Any read that do not have two or more repeat units or in which the divergence rate between repeat units exceeds 25% will be discarded. ecc_finder then uses minimap2 to map these selected reads to a reference genome. Only loci displaying more than two reads coverage are selected. The *value of p* for each base of the genome is calculated assuming a null model with a log-normal distribution given by Genrich. ecc_finder sets the enriched genomic locus as the reference boundary and applies bedtools groupby to calculate the number of tandemly repeated reads, sub-alignments, and boundary coverage (Supplementary Figure 1). Loci covered by a minimum of three reads are kept. Finally, detected loci that are covered for at least 80% of their length are retained.

### Short-Read Pipeline Algorithm Overview

Ecc_finder uses a standard method based on discordant pairs and split reads at the junction to detect reads originating from circular DNA in short-read sequencing data (Supplementary Figure 2). ecc_finder uses BWA-MEM as the default mapping software for short reads because it is more accurate at the basic level alignment, but users can still choose minimap2 with the short-read parameter “sr” to speed up the alignment for large genomes. By grouping by chromosome and read ID, the read alignments are sorted and merged to remove the overlapping reads between a pair, and ecc_finder extracts the read pair information and read pair direction. Properly mapped read pairs with inward-facing tags (“−>, <−”) or single mapped reads (“−> / <−”) are discarded, while discordant read pairs with outward-facing tags (“<−, −>”) are kept. For split reads, ecc_finder then selects read pairs with 3 unique hits on the same chromosome, with orientations suggesting a circular template such as (“−>, <−, −>”) and (“<−, −>, <−”). The enriched genomic sites extracted with Genrich are set as the reference boundaries to group split reads and discordant reads. Only the reads spanning the same boundaries are kept. Loci covered by a minimum of two split reads and one discordant read pair are kept. Finally, the *bona fide* eccDNA-producing loci are defined as regions displaying an even distribution of split and discordant reads (Supplementary Figure 2). In addition, ecc_finder benchmarked BWA-MEM and segemehl to access the accuracy of different short-read aligners. Compared with BWA, segemehl requires more computing time for indexing and aligning, as well as high storage capacity, especially for large genomes (Supplementary Figure 3). In the *Arabidopsis* samples, ecc_finder did not find any difference for eccDNA detection using either segemehl or BWA aligner (Supplementary Figure 3).

### Confidence Score

For short reads, the confidence score of each eccDNA locus is calculated from the number of discordant and splits reads at the locus and the coverage at the locus boundaries. For long reads, the confidence score of each eccDNA locus is calculated from the number of repeat units in each mapping read and the total coverage at the locus boundaries. Users can adjust all parameters to customize confidence score calculation.

### Plant Material and Growth Conditions

Seeds from *Arabidopsis thaliana* WT ecotype Columbia-0 were surface sterilized and sown on 1/2 MS medium [1% sucrose, 0.5% Phytagel (Sigma), pH 5.8], stratified for 2days at 4°C, and grown in a controlled chamber (Percival, United States) at 21°C under long-day conditions (16-h light). Leaf material from 12 individuals was harvested after 2weeks. For heat shock, the *in vitro* plates were exposed at 6°C for 12h and 37°C for 24h and material was extracted after a 24h recovery at 21°C. Swiss winter wheat (*Triticum aestivum* cv. *Arina*) seeds originate from the Agroscope GenBank. Wheat seeds were presoaked in sterilized water overnight and sterilized by a 10min 50°C heat shock. Seedlings were germinated and grown under controlled conditions in a Sanyo MLR-350 growth chamber under long-day conditions 16h (light) at 20°C (day) and 18°C (night) for 4days.

### DNA Extraction

For Arabidopsis, seedlings were collected into one tube immediately snap-frozen in liquid nitrogen and stored at −80°C until DNA extraction, in duplicate. For wheat, three individual seedlings were collected into one tube immediately snap-frozen in liquid nitrogen and stored at −80°C until DNA extraction. For both species, the total DNA was extracted using the CTAB method. Total DNA quantity was measured with a Qubit Fluorometer (Thermo Fisher Scientific).

### eccDNA Enrichment

Genomic DNA (2μg) of each sample was treated with Plasmid-Safe™ ATP-Dependent DNase (Epicentre) according to the manufacturer’s instructions overnight. Following digestion, DNA was precipitated with 0.1 volume of 3M sodium acetate (pH 5.2), 2.5 volumes of ethanol, and 1μl of GlycoBlue^™^ Coprecipitant (Ambion) overnight at −20°C. After centrifugation at 4°C for 1h and washing with 70% ethanol, 100ng of precipitated circular DNA was directly resuspended in the Illustra TempliPhi Sample Buffer and then amplified by random rolling circle amplification using the Illustra^™^ TempliPhi Amplification Kit (GE Healthcare) according to the manufacturer’s instructions. The enriched amplification product was precipitated and debranched using the NEB T7 Endonuclease following the manufacturer’s instructions. For both *Arabidopsis* and wheat samples, 1ng of amplified DNA was used to prepare libraries for Miseq sequencing as in Lanciano et al., 2017. For the wheat samples, after final precipitation, 400ng of DNA was used to prepare an ONT library using the Nanopore Rapid Barcoding Sequencing Kit (SQK-RBK004). DNA was sequenced on a MinION.

### Data and Code Availability

All high-throughput sequencing data generated in this study have been deposited to the European Nucleotide Archive^4^ under the PRJEB46420 project. Source code and test samples for the ecc_finder pipeline are available at https://github.com/njaupan/ecc_finder.

## Results

### Overview of eccDNA Detection Using ecc_finder

**Figure 2:**
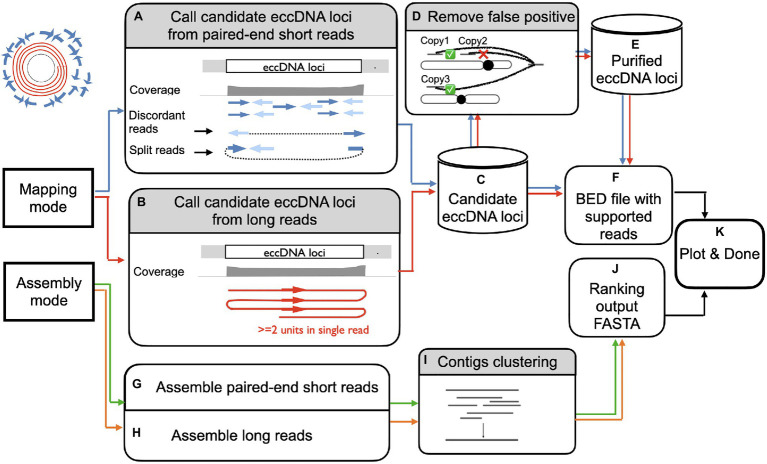
Overview of eccDNA detection using ecc_finder. ecc_finder identifies eccDNA loci from illumina paired -end short reads (SR, *blue/green*) or Nanopore long reads (LR, red/orange) with or without a reference genome. In mapping mode, ecc_finder filters discordant and split reads detected from SR (**A**, *blue*), and filters for more than 2 junctions in a single read from LR (**B**, *red*), the reference genome being provided by the user. Using these filtered reads together with the covergae information, ecc_finder establishes a list of eccDNA candidates **(C)**. ecc_finder further detects false positive eccDNAs originating from repeated loci **(D,E)**. The bed output of a sample is further normalized **(F)** to append multiple samples to create a heat map **(K)**. In assembly mode, ecc_finder assembles SR with a k-mer assembler (**G**, *green*), and/or assembles LR using a repeat unit recognition algorithm (**H**, *orange*). The contigs are then clustered based on highly similarity **(I)**. The output is a fasta file indicating the number of supported reads for each contig **(J)**. Both mapping and assembling mode can be run in parallel to generate a heat map **(K)**.

ecc_finder is designed to analyze eccDNA data generated from eccDNA-seq using Illumina paired-end shorts reads or ONT long reads (Figure 2). Two modes of analysis are proposed: either a mapping mode guided by a reference genome or a *de novo* assembly mode that is reference-free. Both modes can be used on the same data set (hybrid mode). For the Illumina short reads in the mapping mode, ecc_finder first uses BWA (Li and Durbin, 2009; default aligner) to map eccDNA data to a reference genome to detect loci enriched for eccDNA signals (Figure 2A). ecc_finder then detects eccDNA-producing loci based on discordant and split read pairs at junction and then filter by confidence score (Methods). For long reads in the mapping mode, ecc_finder detects circular long reads based on sub-read alignment from tandemly repeated reads (Figure 2B) and further filter by confidence score (Methods). The output of the mapping mode using either Illumina short reads or ONT long reads or a hybrid of both results in a list of candidate loci (Figure 2C).

Taking into account the high similarity of eccDNA-producing repeated loci, ecc_finder then calculates the read distribution of each candidate locus to filter out false positives (Figure 2D). In the end, ecc_finder produces bed files of the coordinates of each eccDNA-producing locus and the corresponding eccDNA sequence (Figure 2E). In addition, for comparative analysis, the bed output of all samples is further normalized to easily implement multiple samples into a final report (Figure 2K).

In the assembly mode, ecc_finder uses the k-mer assembler Spades (Prjibelski et al., 2020) to assemble short reads and the repeat unit recognition and consensus calling tool Tidehunter (Gao et al., 2019) to assemble long reads (Figures 2G,H). Instead of examining the performance of different assemblers, ecc_finder constructs a representative set by clustering the assembled contigs. ecc_finder then uses CD-hit (Li and Godzik, 2006) to self-align contigs to contigs and filter out redundant contigs with 80% similarity (Figure 2I). Finally, the output of ecc_finder is a FASTA sequence file of all contigs ranked by the number of supporting reads (Figure 2J).

### Benchmarking eccDNA Detection Tools Based on Short Reads

**Figure 3:**
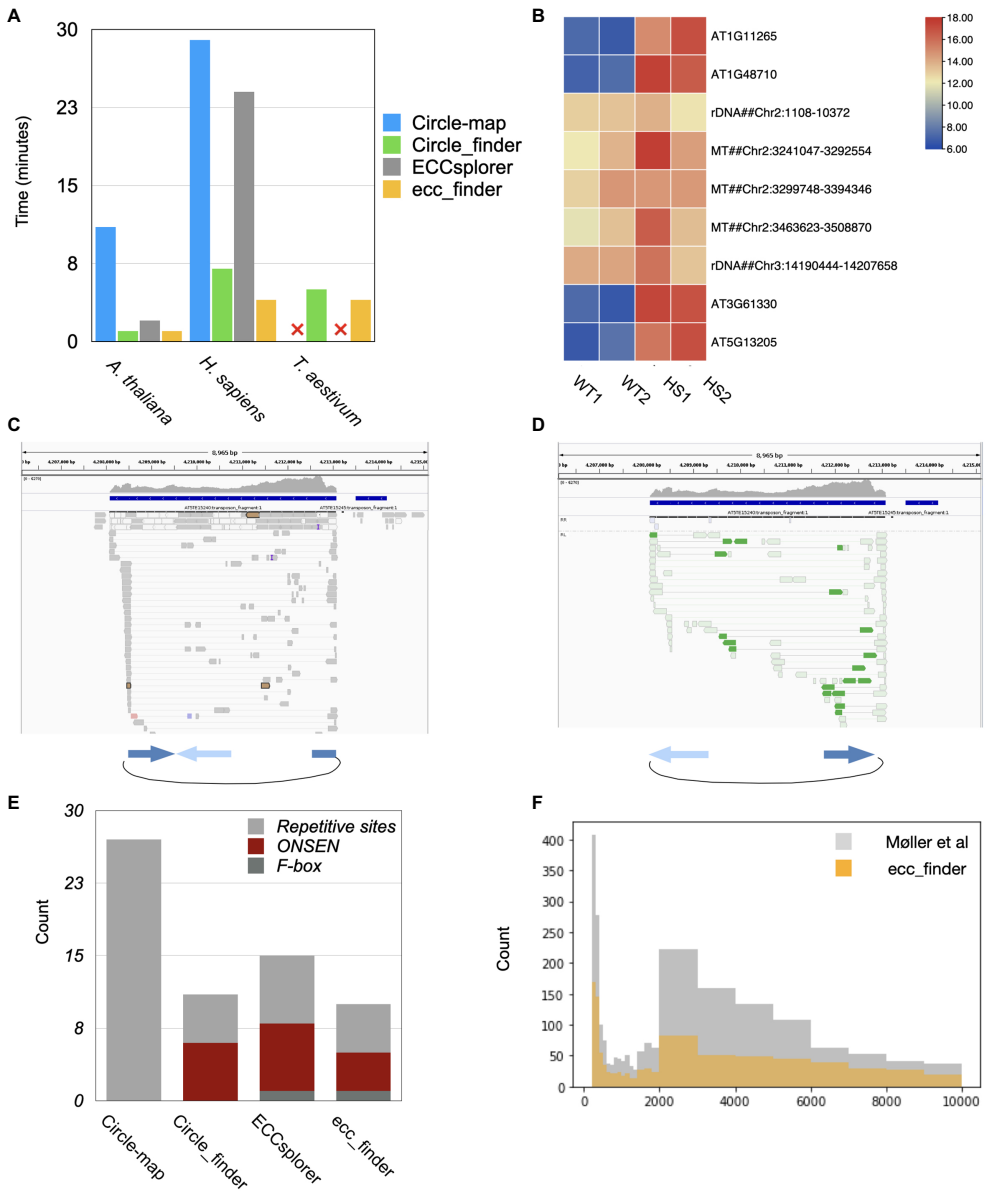
Performance of different eccDNA detection tools using Illumina short reads. **(A)** Real consumed time on three different species using different tools. Circle-map and ECCsplorer failed to process the ecc-DNA-seq data of *T. aestivum* because (1) it ran out of RAM, (2) the csi file produced from samtools index for large genome cannot be used for the next step. **(B)** Heat map of 8 eccDNA sites detected using ecc_finder in the heat-stressed *Arabidopsis* (HS1, HS2) and wild-type Col-0 (WT1, WT2) with two replicates, respectively. Among them, 4 out of 9 correspond to *ONSEN/ATCOPIA78* loci. **(C,D)** Examples of the clear boundaries of split **(C)** and discordant reads **(D)** detected by ecc_finder, supporting the eccDNA form of *ONSEN/ATCOPIA78* in the *Arabidopsis* mobilome-seq data. **(E)** Number of classified genomic sites forming eccDNA detected from different tools in the heat-stressed *A. thaliana* eccDNA-seq data. **(F)** Size distribution of detected eccDNA using ecc_finder compared to original study using the same circular-DNA-enriched dataset (Møller et al., 2018).

In order to evaluate the sensitivity, accuracy, and computational requirements of different eccDNA detection tools, we used public eccDNA data from *Homo sapiens* (NA12878, Møller et al., 2018), and we produced heat-stressed *Arabidopsis thaliana* and common wheat (*Triticum aestivum*) eccDNA-seq data. We selected these species for their diverse genome sizes and for the presence of previously described eccDNAs in the case of *Arabidopsis*. The initial step of eccDNA detection tools corresponds to genome indexing, necessary to speed up the mapping algorithms. Circle-Map, Circle_finder, and ecc_finder (default mode) use BWA to map eccDNA data on the corresponding reference genome, whereas ECCsplorer requires segemehl (Hoffmann et al., 2009). ECCsplorer spent 2.4h indexing the human genome and 16.3h for the wheat genome, which is twice the time compared to BWA. The following comparisons thus excluded indexing and mapping steps, in order to only account for the eccDNA detection step. Among all tools, ecc_finder greatly improved the computational time and performed faster on all data sets, followed by Circle_finder, and ECCsplorer (Figure 3A). The Circle-Realign step of Circle-map is the most time-consuming one, and it cannot process index files created by BWA for very large genomes (such as wheat). ECCsplorer is the only up-to-date automated pipeline that can detect eccDNA and establish consensus sequences for non-model species. Unfortunately, it failed to process our wheat eccDNA data because the tools it implemented ran out of memory and disk storage on a cluster with 96 CPUs and 496GB RAM (segemehl produced 200G for indexing the wheat genome). Therefore, when considering computation performance, ecc_finder is one of two options to solve the problem of eccDNA analysis in large genomes.

We then evaluated the eccDNA detection accuracy of all tools. ecc_finder filters eccDNA-producing loci not only by the number of split and discordant reads, their alignment, and orientation but also by genomic enrichments to remove noisy signals and optimize redundancy. In heat-stressed *Arabidopsis* samples, ecc_finder detected 4 eccDNA generating loci corresponding to the active copies of the *ONSEN/ATCOPIA78* TE (Figure 3B; Sanchez et al., 2017). ecc_finder further identified known eccDNAs originating from repeats: a 9321bp region located on chromosome 2 (Chr2:1029–10,350) and a 18433bp region located on chromosome 3 (Chr3:14190444–14207658) corresponding to rDNA (Cloix et al., 2000; Abou-Ellail et al., 2011). As expected, ecc_finder also detected a region encompassing 245.8kb on chromosome 2 (Chr2:3234927–3294252, Chr2:3297349–3,401,635, Chr2:3424305–3,453,213, and Chr2:3456196–3509451) and corresponding to mitochondrial DNA integrated into the nuclear genome (Saccone et al., 2000).

All detected eccDNAs had previously been validated, showing the accuracy of ecc_finder in identifying eccDNA-producing loci. The clear eccDNA sequence boundaries of ecc_finder output are also a specificity of this tool (Figures 3C,D). By comparison, ECCsplorer detected 7 *ONSEN* producing loci, 2 of 7 being incomplete and corresponding to false positives, Circle_finder detected 6 *ONSEN* producing loci, 2 of them being false positive, while Circle-map did not detect any *ONSEN* eccDNA (Figure 3E; Supplementary Figures 4, 5; Supplementary Table 1).

We then tested ecc_finder accuracy in detecting eccDNAs in human and wheat eccDNA-seq data. By default, ecc_finder removes circles smaller than 100bp to reduce the noise coming from satellites. The eccDNA size distribution in the human data set indicated that ecc_finder detected a smaller set of eccDNA, but remained similar to the size found in the original study (Figure 3F). ECCsplorer and Circle_finder also detected a smaller set, while Circle-map gave similar circle size ranges (Supplementary Figure 6). For the wheat data set, given that over 80% of the wheat genome contains TEs (Wicker et al., 2018), eccDNA detection is challenging. ecc_finder detected 600 eccDNA-producing loci in the wheat eccDNA-seq (Illumina data) filtering by at least 10 split reads and 5 discordant reads, with 95.5% of these loci also being identified by Circle_finder.

### Detection of eccDNA in Wheat Using Nanopore Long Reads

**Figure 4:**
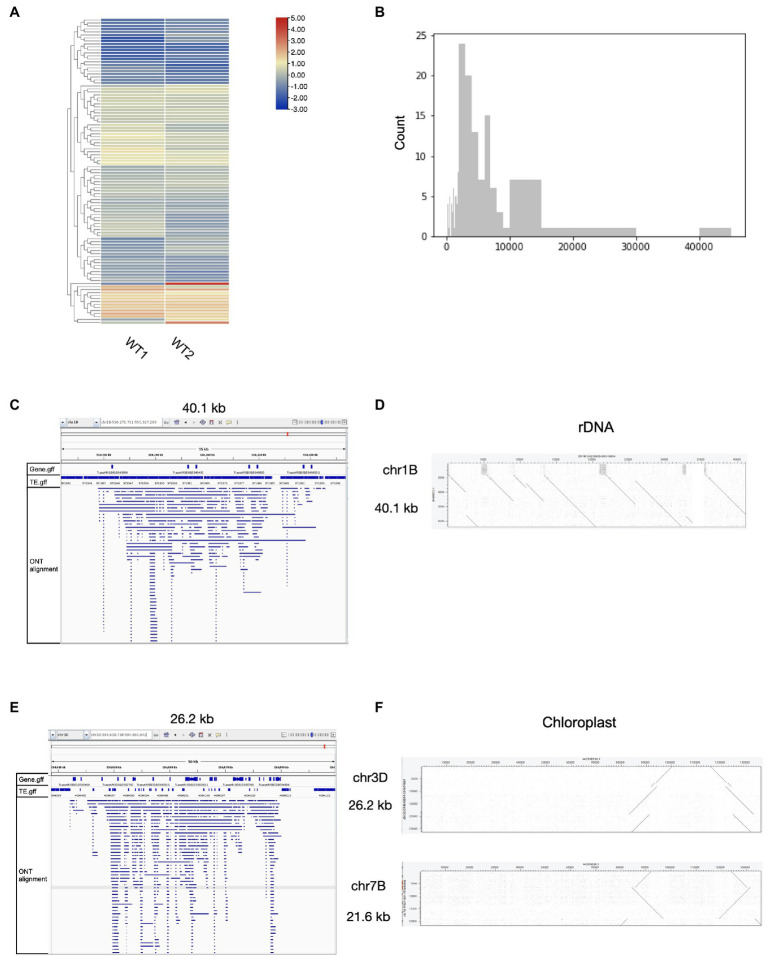
Corresponding origins of eccDNAs loci detected by ecc_finder in wheat using mobilome-seq with Nanopore long reads. **(A)** Heat map of the 161 eccDNA sites detected using ecc_finder in the wheat wild-type (WT1, WT2) with two replicates, respectively. **(B)** Size distribution of the 161 detected eccDNAs. **(C)** Read distribution of the largest eccDNA-producing locus (40.1kb) mapping to the reference genome. **(D)** Dot plot of the largest ecc-DNA-producing locus (26.2kb) mapped to the reference genome. **(E)** Read distribution of the second largest eccDNA-producing locus (26.2 kb) mapped to the reference genome. **(F)** Dot plot of two eccDNA-producing loci (26.2kb and 21.6kb) and chloroplast genome, respectively.

We then tested the performance of ecc_finder on wheat ONT eccDNA-seq data. ecc_finder detected 161 eccDNA-producing sites in two replicates (Figure 4A). These loci were distributed over all the 21 chromosomes, with eccDNA sizes ranging from 100bp (detection threshold) to 40.1kb (Figure 4B). We further characterized the output of ecc_finder and confirmed that the eccDNA-producing loci corresponded to rDNA, chloroplast DNA, and repetitive sequences. For example, the largest eccDNA-producing locus on chromosome 1B is 40.1kb long and covers an 18S rDNA gene cluster (Figure 4C). The second and third largest loci (26.2kb on chromosome 1D and 21.6kb on chromosome 7B) are 99% identical to the chloroplast genome (Figure 4D). Overall, these findings are consistent with the observations we made in *Arabidopsis*.

### Detection of eccDNA in Short-Read Genomic Data Without eccDNA Enrichment

EccDNAs have recently been identified from genomic and/or ATAC-seq data in mammal samples (Turner et al., 2017; Wu et al., 2019; Kumar et al., 2020), without prior enrichment for circular DNA. We have tested ecc_finder on genomic data using the tumor sample GBM39 sequenced by low coverage whole-genome sequencing (50bp paired-end reads; Turner et al., 2017). ecc_finder successfully detected 95 discordant reads at the junction of the 1.29Mb eccDNA (data not shown), which is also consistent with the results of Wu et al., (2019). However, ecc_finder was unable to construct the final structure of this large eccDNA because no split read could be detected. Therefore, in its mapping mode, ecc_finder will output the bed file containing the numbers of split and discordant reads for any genomic data. However, the peak calling will not be effective because of the lack of enrichment. We have not tested ecc_finder on ATAC-seq data but a recent method for this specific type of data has been described (Kumar et al., 2020).

## Discussion

EccDNA-producing loci can be repeated in the genome. However, current tools do not take into account the repeated nature of these loci, and the detected loci can thus be redundant, notably for TEs, rDNA, and satellites. In this case, identifying the exact locus producing eccDNA can be challenging. For a given family of long terminal repeats (LTR) retrotransposons producing eccDNA for instance, all copies belonging to the same family and sharing the same LTR sequences will produce alignments of split and discordant reads at their boundaries. Only the copies producing *bona fide* eccDNA will thus display an even distribution of split and discordant reads throughout their internal region. ecc_finder implemented this step in its detection of eccDNA-producing loci in order to improve the detection of eccDNAs. Additionally, ecc_finder enables the use of eccDNA long-read sequencing data that is likely to become the standard in the coming years.

## Conclusion

Although eccDNA was known for decades in yeasts, plants, and animals, growing evidence in recent years suggests that this peculiar form of DNA plays a role in rapid adaptation, for instance in cancer cells (Kumar et al., 2017; Verhaak et al., 2019; Kim et al., 2020; Wang et al., 2021) or herbicide resistant plants (Koo et al., 2018), by promoting overexpression and alternate epigenetic state of a selected set of genes. Characterizing the full repertoire of eccDNA is becoming crucial, and several protocols are available to enrich a DNA sample for eccDNA and sequence it with short or long reads. We believe that ecc_finder that was developed here will facilitate the downstream bioinformatic analysis of these data sets, notably for ONT long reads, and accelerate the discoveries linked to eccDNA biology in many species, including the ones with the largest genomes and high transposable element content.

## Data Availability Statement

Publicly available datasets were analyzed in this study. This data can be found at: European Nucleotide Archive (ENA) repository under project number PRJEB46420 (https://www.ebi.ac.uk/ena/browser/view/PRJEB46420).

## Author Contributions

PZ produced mobilome-seq data, wrote the bioinformatic scripts, analyzed data, and wrote the manuscript. HP produced and analyzed mobilome-seq data and wrote the manuscript. CL produced mobilome-seq data. EB analyzed data and wrote the manuscript. MM designed the experiment, analyzed data, and wrote the manuscript. All authors contributed to the article and approved the submitted version.

## Funding

MM is supported by a grant from the French National Agency for Research (ANR-13-JSV6-0002 “*ExtraChrom”*). This study is set within the framework of the “Laboratoire d’Excellence (LABEX)” TULIP (ANR-10-LABX-41) and of the “Ecole Universitaire de Recherche (EUR)” TULIP-GS (ANR-18-EURE-0019). PZ, MM, and EB are members of the European Training Network “*EpiDiverse*” that received funding from the EU Horizon 2020 program under Marie Skłodowska-Curie grant agreement No 764965. HP is supported by China Scholarship Council (CSC) Grant (201806990012). EB and HP received funding from the European Research Council (ERC) under the European Union’s Horizon 2020 research and innovation program (Grant agreement No. 725701, BUNGEE). MM and EB are supported by a grant from the Agence Nationale de la Recherche and Fonds National Suisse (ANR-21-PRCI-CE02, FNS 310030E_205554 “CropCircle”).

## Conflict of Interest

The authors declare that the research was conducted in the absence of any commercial or financial relationships that could be construed as a potential conflict of interest.

## Publisher’s Note

All claims expressed in this article are solely those of the authors and do not necessarily represent those of their affiliated organizations, or those of the publisher, the editors and the reviewers. Any product that may be evaluated in this article, or claim that may be made by its manufacturer, is not guaranteed or endorsed by the publisher.

## Footnotes

^2^ https://github.com/crimBubble/ECCsplorer

^3^ https://github.com/jsh58/Genrich

^4^ https://www.ebi.ac.uk/ena
