# Supplementary material for: ecc_finder: A Robust and Accurate Tool for Detecting Extrachromosomal Circular DNA From Sequencing Data
Source: Front Plant Sci. 2021 Dec 1;12:743742. doi: 10.3389/fpls.2021.743742 (PMC8672306; doi:10.3389/fpls.2021.743742)
Supplement: Supplementary file 1 [file Presentation_1.pdf]

Zhang P, Peng H, Llauro C, Bucher E and Mirouze M (2021)
ecc_finder: A Robust and Accurate Tool for Detecting Extrachromosomal Circular DNA From Sequencing Data.
Front. Plant Sci. 12:743742. doi: 10.3389/fpls.2021.743742

Supporting Information

A. Removing satellites by query read length and aligned length.

B. Detecting tandem repeat pattern from long read (minimum 2 repeat units).

C. Calculating p-value for peak calling (maximum false discovery rate 1%).

D. Calculating confidence score.

Minimum 2 sub-alignments in the same orientation

Minimum tandemly repeated read number (>=3)

Minimum boundary coverage =

aligned bases / boundary bases for each tandemly repeated read (80%)

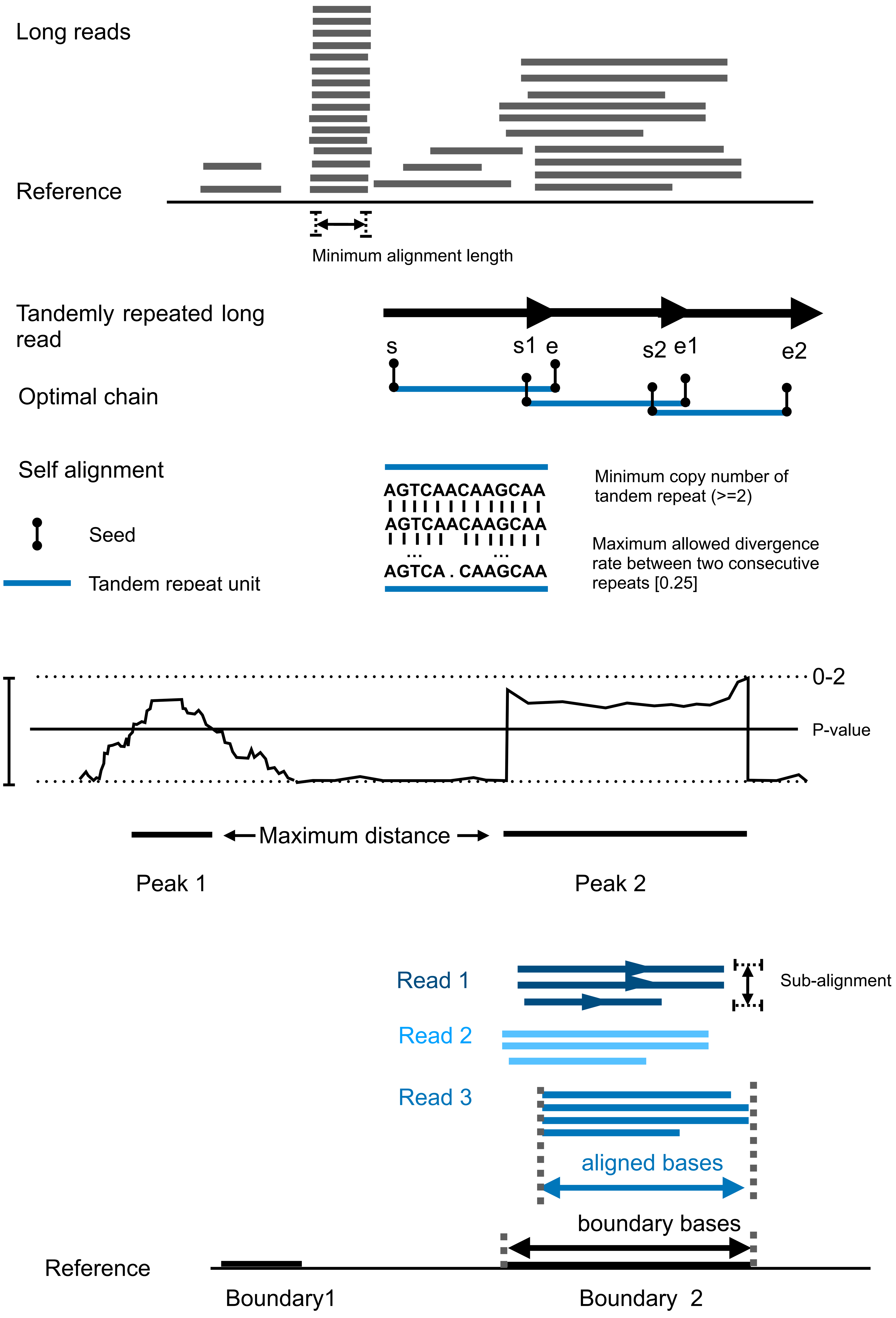

Supplementary Figure 1. Overview of the long-read pipeline algorithm of ecc_finder.

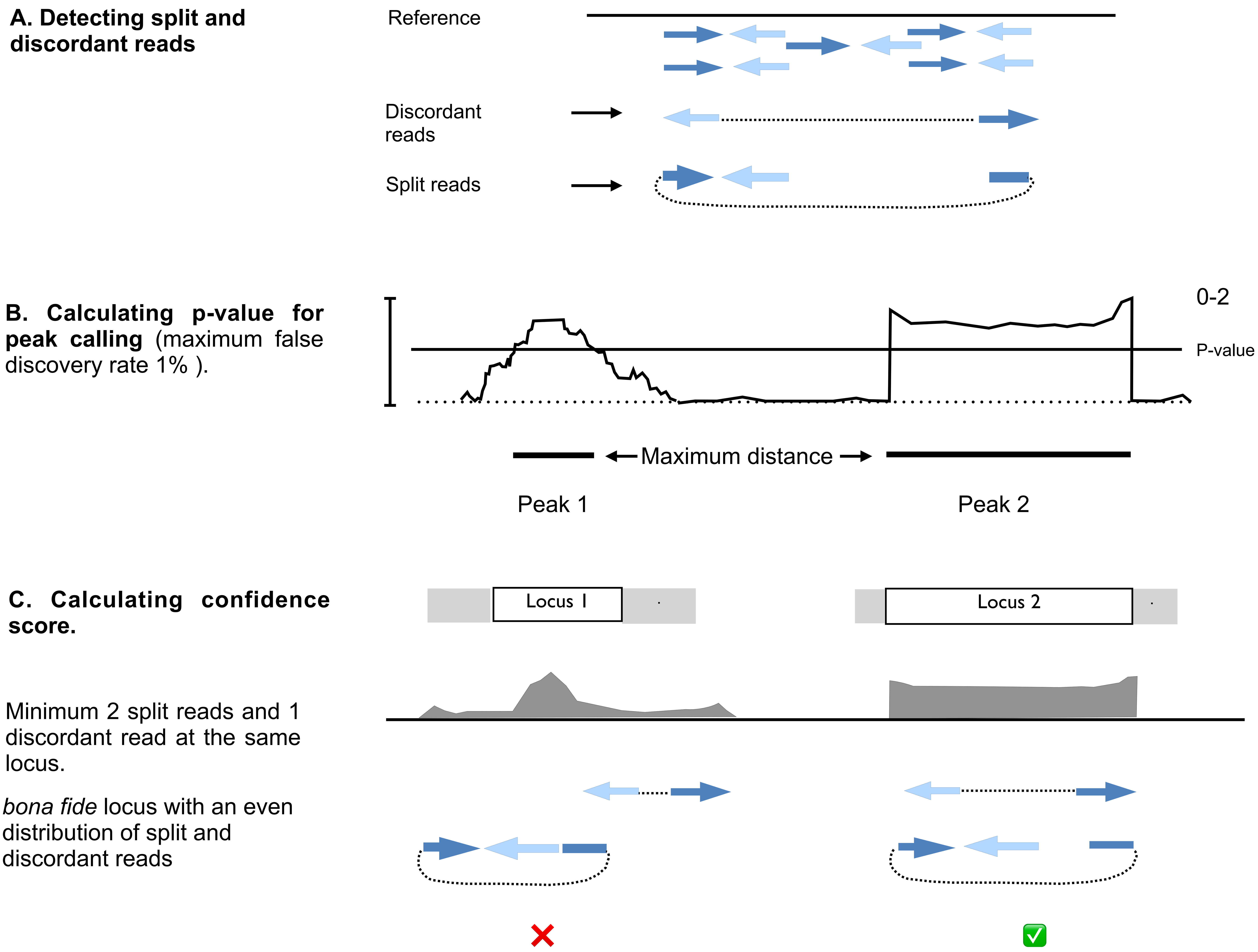

Supplementary Figure 2. Overview of the short-read pipeline algorithm of ecc_finder.

A

		A. thaliana	*H. sapiens*	*T. aestivum*
Computational time (index+ align)	BWA	1m 51s + 1m 25s	1h 6m 49s + 5m 11s	9h 30m 3s + 18m 44s
	Segemhel	2m 46s + 22m 38s	2h 24m 47s + 3h 33m	>16h out of RAM
Requried capacity (Byte)	BWA	202 M	5.3 G	25 G
	Segemhel	1.6 G	45 G	> 200G

B

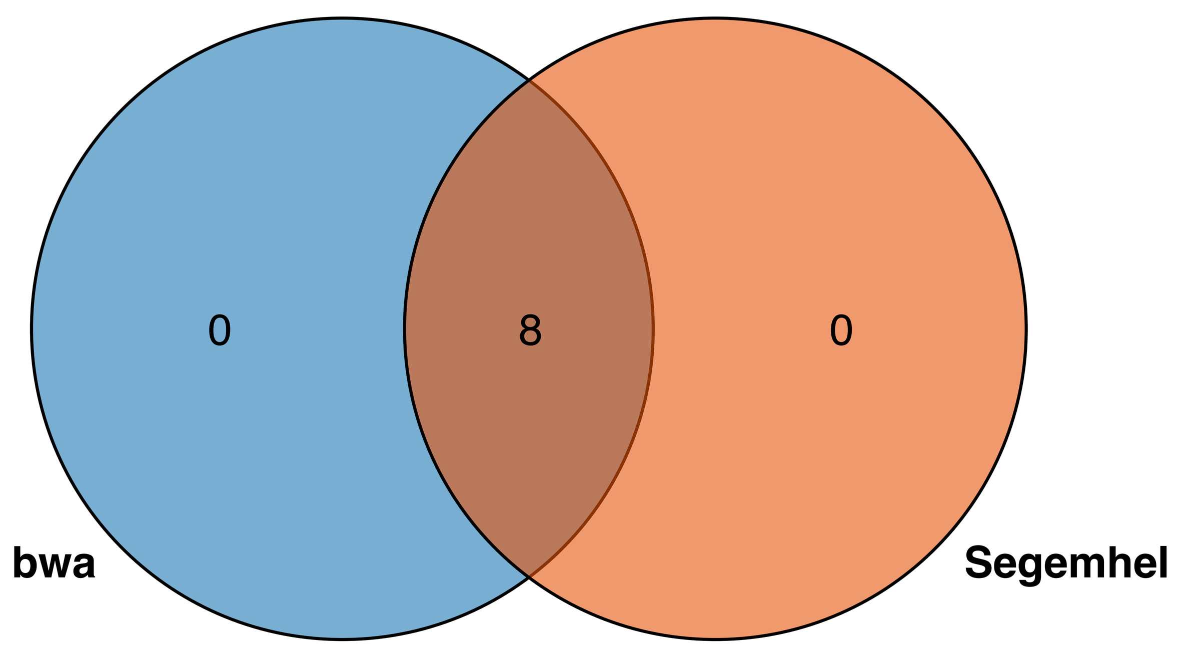

C

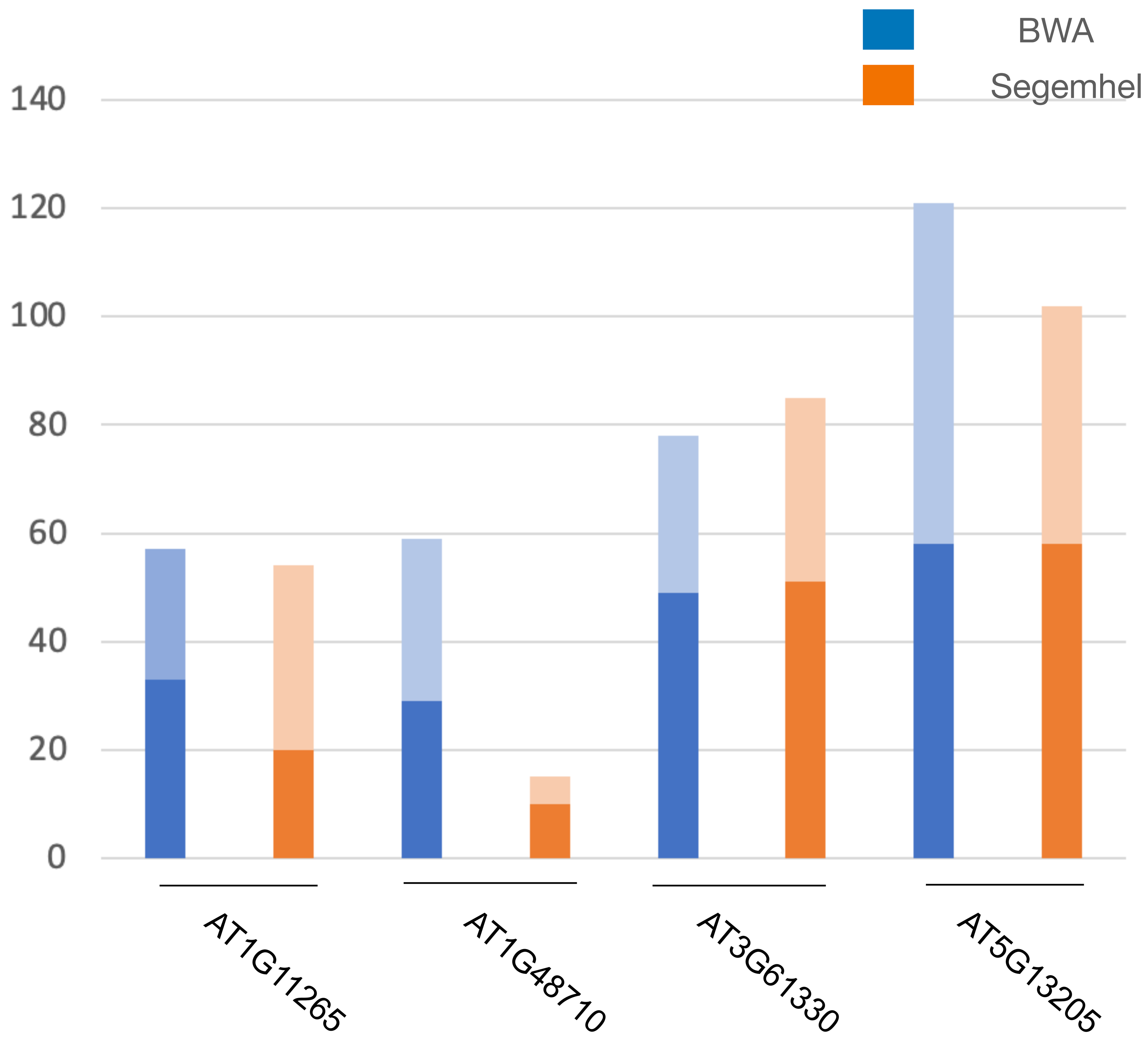

Supplementary Figure 3. Performance of ecc_finder using different aligners on Illumina short reads. Segemhel failed to process the eccDNA-seq data of *T. aestivum* because it ran out of RAM and disk storage on a cluster with 96 CPUs and 496 GB RAM (200G produced to index the wheat genome). **(A)** Comparison of computational time and required capacity for two aligners. **(B)** Intersection of eccDNA loci detected by ecc_finder using BWA and Segemhel in the heat-stressed *Arabidopsis*; **(C)** Number of split reads and discordant reads (light colour) detected by ecc_finder using BWA and Segemhel in the heat-stressed *Arabidopsis* at 4 *ONSEN* loci.

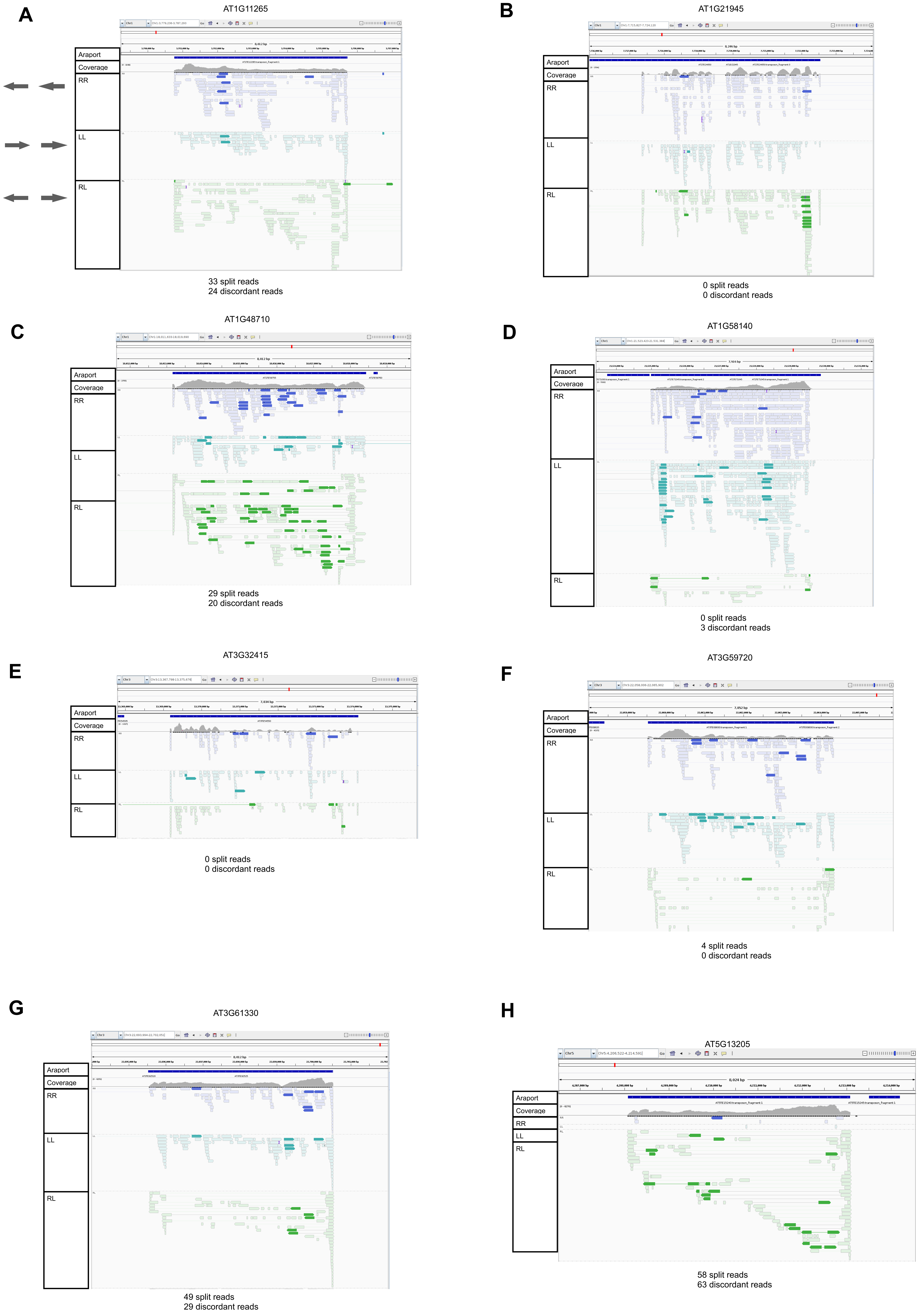

Supplementary Figure 4. Distribution of split and discordant reads at the loci of the 8 individual *ONSEN/ATCOPIA78* family members in the heat-stressed *Arabidopsis*. (A-H) Paired reads were interpreted by orientations. RR & LL: Illumina sequence read pairs align in the same orientation with respect to reference. RL: Illumina sequenced read pairs align in an outward-facing order with respect to reference that indicate discordant reads.

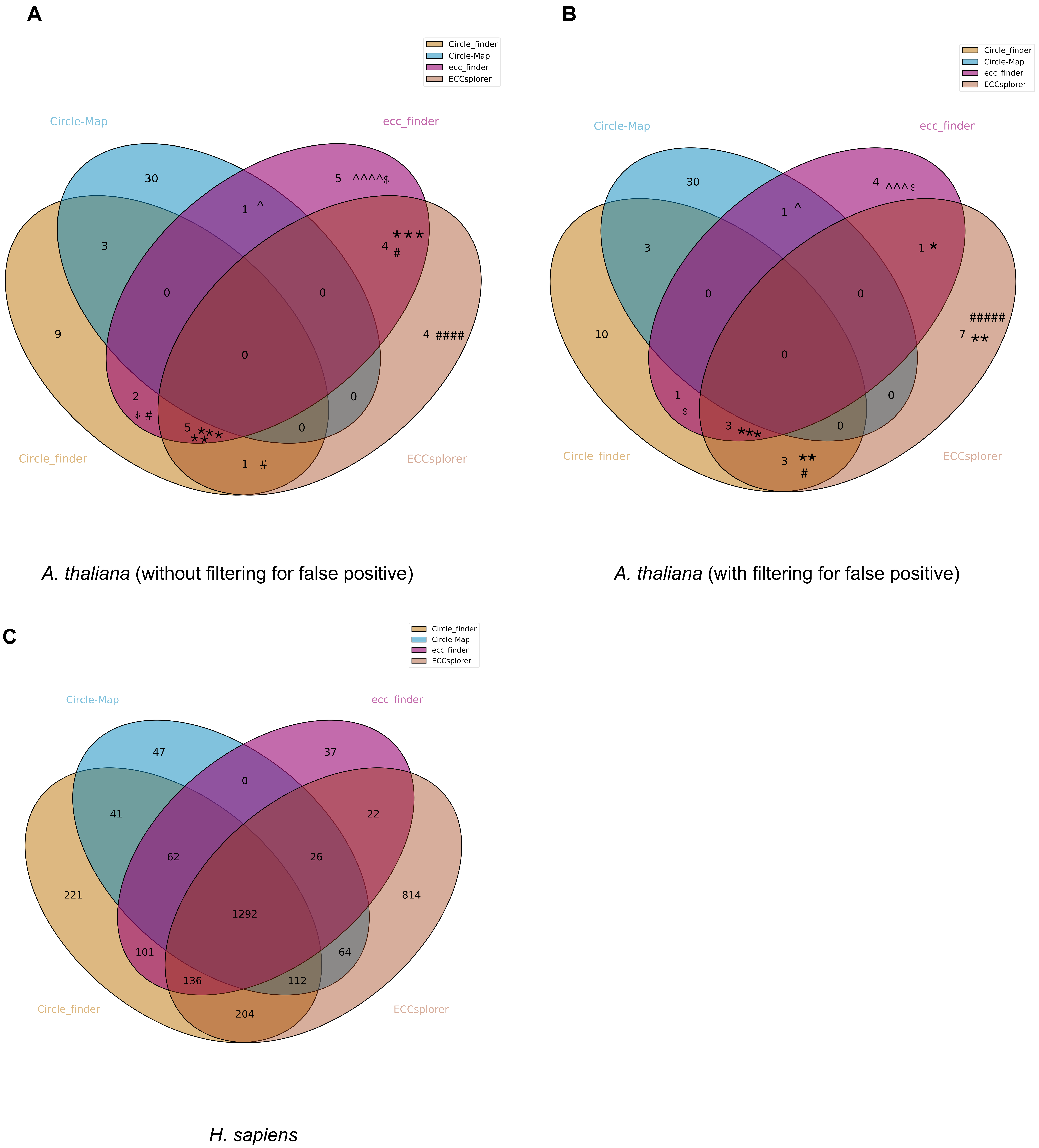

Supplementary Figure 5. Comparison of different eccDNA detection tools using Illumina short reads eccDNA-seq datasets. (A) eccDNA detected in *Arabidopsis* heat stress sample. Here we show the ecc_finder output without the automatic filtering for false positives. The detected eccDNA originating from *ONSEN* (*), rDNA ($), 180bp tandem centromeric repeats (#), and mitochondrial DNA (^) are highlighted. **(B)** eccDNA detected in *Arabidopsis* heat stress sample, applying (for ecc_finder) the automatic filter to remove false positives. Note that only 3 eccDNA producing loci are kept for *ONSEN*. See Supplementary Table 1 for detailed output description. **(C)** eccDNA detected in the human dataset.

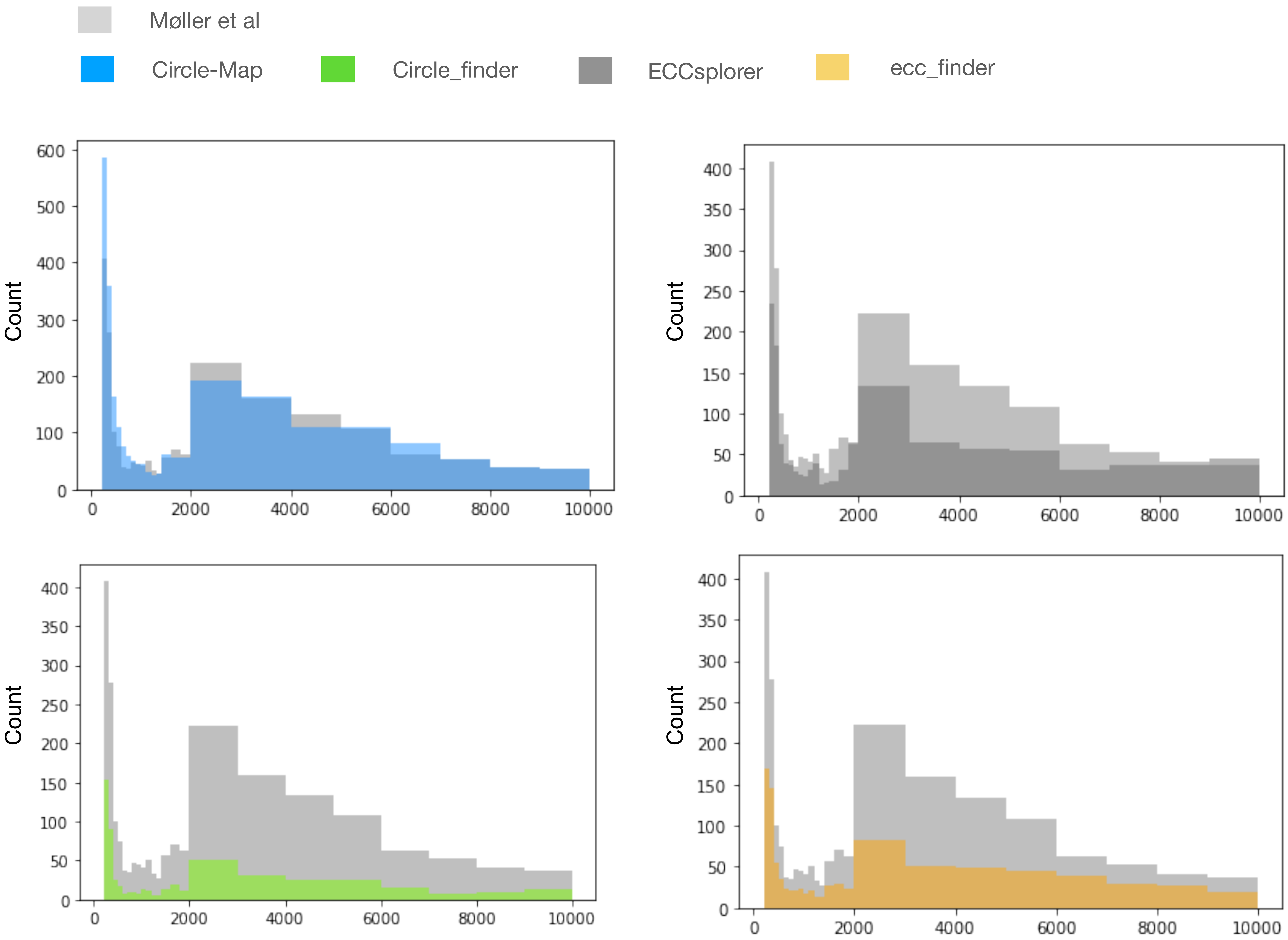

Supplementary Figure 6. Size distribution (in bp) of the eccDNA detected from different tools based on the same circular DNA enriched muscle dataset from Møller et al., 2018.

Tool	Chromosome	Start position	End position	Annotation
ecc_finder	Chr1	3780760	3785723	ONSEN
	Chr1	18012790	18018425	*ONSEN*
	Chr2	1029	10350	rDNA
	Chr2	3234927	3294252	MT
	Chr2	3297349	3401635	MT
	Chr2	3424305	3453213	MT
	Chr2	3456196	3509451	MT
	Chr3	14190340	14208773	rDNA
	Chr3	22695562	22700528	*ONSEN*
	Chr5	4207752	4213091	*ONSEN*
ECCsplorer	Chr1	3358109	3360689	*ONSEN*
	Chr1	3780762	3785720	*ONSEN*
	Chr1	7720244	7722108	*ONSEN*
	Chr1	18013160	18018120	*ONSEN*
	Chr1	21524994	21530008	*ONSEN*
	Chr2	3606745	3627625	cen
	Chr3	13587605	13587783	cen
	Chr3	13588991	13592453	cen
	Chr3	22056556	22064330	*ONSEN*
	Chr3	22695565	22700523	*ONSEN*
	Chr4	3952315	3953647	cen
	Chr5	4208082	4213086	*ONSEN*
	Chr5	11723578	11724366	cen
	Chr5	11732461	11734380	cen
Circle_finder	Chr1	3780760	3785723	*ONSEN*
	Chr1	12234370	12234859	AT1G33750
	Chr1	18013158	18018425	*ONSEN*
	Chr1	19669405	19670288	AT1G52820
	Chr1	21524990	21529669	*ONSEN*
	Chr1	27795290	27795772	AT1G73930
	Chr3	5478612	5479317	AT3G16170
	Chr3	13369172	13374108	*ONSEN*
	Chr3	14194495	14204731	rDNA
	Chr3	22059531	22064331	*ONSEN*
	Chr3	22453376	22454668	*ONSEN*
	Chr4	1750742	1753587	*ATCOPIA95*
	Chr4	1753818	1754282	*ATCOPIA49*
	Chr4	3950517	3953850	cen
	Chr4	6075920	6076784	intergenic
	Chr4	6076951	6077910	intergenic
	Chr4	7684033	7684692	AT4G33090
	Chr5	4208079	4213089	*ONSEN*
	Chr5	4649552	4650534	AT5G14420
	Chr5	5215112	5215691	AT5G15980

Supplementary Table 1. Output of different eccDNA detection tools (without circle-map) on Illumina short reads in the *Arabidopsis* heat stress sample.
